# The peri / postoperative analgesic effect of intravenous paracetamol in dogs

**DOI:** 10.18849/ve.v9i2.683

**Published:** 2024-04-04

**Authors:** Laura Mckay+

**Keywords:** CANINE, DOGS, INTRAVENOUS PARACETAMOL, PERIOPERATIVE ANALGESIA, POSTOPERATIVE ANALGESIA

## Abstract

**PICO question:**

In healthy dogs undergoing a surgical procedure, is there improved pain control in dogs receiving intravenous paracetamol in the peri / postoperative period compared to dogs not receiving intravenous paracetamol?

**Clinical bottom line:**

**Category of research:**

Treatment.

**Number and type of study designs reviewed:**

Three randomised, controlled, and blinded studies. Two studies directly address the PICO question whereby postoperative pain assessment was clinically evaluated following intravenous (IV) paracetamol. The third study addressed the question to a lesser extent, whereby the impact on the sevoflurane minimum alveolar concentration (MAC) reduction in response to noxious stimuli was assessed following the administration of IV paracetamol.

**Strength of evidence:**

Weak.

**Outcomes reported:**

The findings of the first two studies presented appear to directly contradict each other. The first study demonstrated a reduction in pain in all groups and found no differences in analgesia between IV paracetamol and other non-steroidal anti-inflammatories drugs (NSAIDs), while the second study reported no analgesia effects from IV paracetamol and was terminated prematurely because a high number of dogs required rescue analgesia. The first study reported sufficient analgesic effects of IV paracetamol and the second study reported no analgesia effects of IV paracetamol. Both were blinded, randomised, controlled studies and directly addressed the PICO question in relation to the peri / postoperative analgesic effects of IV paracetamol. However, their methods and sample sizes were very different. The third study did not demonstrate a clinically relevant sevoflurane MAC reduction after IV paracetamol in dogs.

**Conclusion:**

At present, there is limited and weak evidence to suggest that IV paracetamol provides peri / postoperative analgesia in dogs. However, further studies are required to better assess its efficacy, its duration of action, and the appropriate doses that are necessary to reach therapeutic plasma levels. The reduced incidence of side effects at the currently recommended doses could support its peri / postoperative use, where NSAIDs use is contraindicated.

## How to apply this evidence in practice

The application of evidence into practice should take into account multiple factors, not limited to: individual clinical expertise, patient’s circumstances and owners’ values, country, location or clinic where you work, the individual case in front of you, the availability of therapies and resources.

Knowledge Summaries are a resource to help reinforce or inform decision-making. They do not override the responsibility or judgement of the practitioner to do what is best for the animal in their care.

## Clinical scenario

A geriatric dog diagnosed with stage two renal disease is presented for ovariohysterectomy. While non-steroidal anti-inflammatories drugs (NSAIDs) are commonly used to produce postoperative analgesia in healthy dogs, their administration in this specific case might be contraindicated because of the renal disease. Inhibiting the cyclooxygenase enzymes and the production of prostaglandins, NSAIDs increase the risk of kidney ischaemia and damage during periods of hypotension (KuKanich et al. 2012). Therefore, would intravenous (IV) paracetamol be a good alternative to NSAIDs to provide postoperative analgesia with low incidences of adverse side effects?

## The evidence

The results of three randomised, controlled, and blinded studies are included. While two of those studies (Hernández–Avalos et al., 2020; and Leung et al., 2021) directly addressed the PICO question, the third study (González-Blanco et al., 2020) evaluated the effect of intravenous (IV) paracetamol on sevoflurane minimum alveolar concentration (MAC). The evidence is contradictory in some areas and, overall, is limited and weak.

## Summary of the evidence

**Hernández-Avalos et al. (2020):** 

Population	Client-owned dogs undergoing elective ovariohysterectomy.
Sample Size	30 dogs.
Intervention Details	Dogs were divided into three treatment groups; 10 dogs receiving paracetamol (15 mg/kg intravenous [IV]), 10 dogs receiving carprofen (4 mg/kg IV) and 10 dogs receiving meloxicam (0.2 mg/kg IV) 30 minutes prior to surgery. Paracetamol was administered every 8 hours and non-steroidal anti-inflammatories drugs (NSAIDs) every 24 hours. All three treatments were continued for 48 hours. A technician administered each treatment and the evaluator was blinded to animals’ group assignments. Postoperative pain was evaluated using two different pain scales; the Dynamic Interactive Visual Analog Scale (DIVAS) and Pain Scale of the University of Melbourne (UMPS). Pain scores were assessed by the same veterinary anaesthetist at 1, 2, 4, 6, 8, 12, 16, 20, 24, 36, and 48 hours following surgery.
Study design	Blinded, randomised, controlled study.
Outcome studied	Postoperative analgesia, cardiorespiratory parameters, liver and renal function were compared between groups.
Main findings (relevant to PICO question)	Postoperative pain decreased throughout the 48 hour period in all three groups. No statistically significant differences in pain scores was found between treatment groups. Rescue analgesia was administered to four dogs in the postoperative period; two dogs in the carprofen group and one dog in the paracetamol and meloxicam group.
Limitations	The pain scoring systems utilised are not validated in dogs. As assessment was performed by the same observer but using two different scoring systems therefore, one scale could have affected the results of the second one. Plasma concentrations of paracetamol were not assessed. The sample size calculation was not adequately explained in the manuscript and 10 dogs per group is a very limited sample, therefore type II statistical error cannot be ruled out. A negative control group was not included.

**Leung et al. (2021):** 

Population	Client-owned healthy dogs undergoing elective ovariohysterectomy.
Sample Size	14 dogs.
Intervention Details	Following routine ovariohysterectomy, a separate individual not involved in pain assessment prepared and disguised the drug and placebo to maintain blinding. Seven dogs were allocated to receive paracetamol (20 mg/kg intravenous [IV]) and seven dogs the equivalent volume of saline. Pain was assessed at 10, 20, 40, 60, 120, and 180 minutes following tracheal extubation using the Short Form of the Glasgow Composite Pain Scale. Rescue analgesia administered in the postoperative period consisted of methadone (0.2 mg/kg IV) and meloxicam (0.2 mg/kg SC) if the intervention threshold was exceeded on the aforementioned pain scale.
Study design	Prospective, blinded, randomised clinical trial.
Outcome studied	To investigate the analgesic effects of paracetamol postoperative intravenous administration in dogs undergoing ovariohysterectomy. Serial plasma analysis of paracetamol levels was assessed concurrently.
Main findings (relevant to PICO question)	3/7 dogs in both groups required rescue analgesia at 20 minutes from tracheal extubation. 4/7 dogs in both groups required analgesia at 60 minutes after tracheal extubation. Overall, 10/14 dogs required rescue analgesia: 4/7 (57.1%) in the paracetamol group and 6/7 (85.7%) in the saline group. Due to the high number of dogs requiring rescue analgesia the study was terminated prematurely at 14 dogs, the original number of dogs planned for the study was 34 dogs. No difference in postoperative pain and the need of rescue analgesia was found between paracetamol (20 mg/kg IV) and saline in dogs undergoing ovariohysterectomy.
Limitations	According to the initial sample size calculation, 14 dogs per group would have been necessary to show a difference of 45% in the rescue analgesia requirement between the group. However, only seven dogs per group were included as the study was prematurely terminated because it was deemed unethical due to the high percentage of dogs requiring rescue analgesia. 10/14 (71.4%) dogs needed rescue analgesia overall). Because of the small sample size, a type II statistical error cannot be categorically excluded.

**González-Blanco et al. (2020):** 

Population	Healthy adult laboratory Beagle dogs.
Sample Size	Seven dogs.
Intervention Details	The dogs were anaesthetised on two separate occasions (2 weeks apart). The treatment was a single intravenous (IV) injection of 15 mg/kg of paracetamol or the equivalent volume of saline administered over 15 minutes. Following a 20 minute equilibrium period, minimum alveolar concentration (MAC) of the gaseous agent sevoflurane was assessed by applying a noxious stimulus using intestinal forceps clamped to the first ratchet lock for 60 seconds on the tail. Plasma levels of paracetamol were analysed 2 minutes following treatment and prior to termination of the gaseous agent.
Study design	Prospective, randomised, blinded, crossover study.
Outcome studied	To determine the effect of a single IV injection of paracetamol on the MAC of sevoflurane in response to noxious mechanical stimuli in dogs.
Main findings (relevant to PICO question)	Paracetamol (15 mg/kg IV) did not reduce the sevoflurane MAC in Beagle dogs.
Limitations	Small sample size. There are limited studies to determine plasma concentrations and duration of action in IV paracetamol in dogs; therefore, the therapeutic range may be insufficient to cause a reduction of MAC. It was noted that low levels of paracetamol were measured in the saline group, explained by the cross-contamination of the phenolic compound found in propofol, which was the induction agent used in both groups. The study did not detail how it was blinded.

## Appraisal, application and reflection

In the UK, a product containing paracetamol and codeine is licensed in dogs as an oral formulation (Pardale- VTM). In particular, 33 mg/kg of paracetamol and 0.75 mg/kg codeine repeated every 8 hours can be administered to dogs for up to 5 days. In dogs undergoing different types of surgeries, the postoperative analgesic effect of a paracetamol-codeine administered every 8 hours was not inferior to meloxicam (Pacheco et al., 2020). The aforementioned studies in the summary of evidence utilise a human formulation of intravenous paracetamol. There is no veterinary licensed equivalent.

According to the BSAVA Formulary (BSAVA, 2020), paracetamol should be administered at 10–20 mg/kg intravenously (IV). While a pharmacokinetic study was performed using this dose range in Beagle and Galgo español dogs, the variable therapeutic range observed between the two dog breeds made the authors conclude that further investigations were warranted to better understand the paracetamol pharmacokinetic properties in the context of anti-nociception in dogs (Serrano-Rodríguez et al., 2019).

Despite that the results of Hernández-Avalos et al. (2020) and Leung et al. (2021) appear to contradict each other, the methodology used makes a comparison between them difficult. Both of them are blinded, randomised, controlled studies, and they tried to address the PICO question in relation to the postoperative analgesic efficacy of paracetamol. In both studies, dogs undergoing ovariohysterectomies were used. Still, the intraoperative analgesic technique employed was different and could have affected the level of postoperative pain and, therefore, the effectiveness of paracetamol. While in Leung et al. (2021), preoperative pethidine was administered as an analgesic, a fentanyl constant rate infusion was used by Hernández-Avalos et al. (2020).

Pethidine is a synthetic opioid and has been shown to provide a dose-dependent effect in dogs, with 3.5 mg/kg intramuscularly (IM) providing 90 minutes of analgesia (Waterman & Kalthum, 1989). According to Lascelles et al. (1997), pethidine (5 mg/kg IM) is an effective but short-acting analgesic in dogs undergoing ovariohysterectomy. Vettorato & Bacco (2011) showed that pethidine (5 mg/kg IM) produced postoperative analgesia up to 4 hours in dogs undergoing ovariectomy or ovariohysterectomy.

The study from Hernández-Avalos et al. (2020), a perioperative fentanyl constant rate infusion (5 mg/kg/hr IV) was administered. Fentanyl is a potent, short-acting opioid and has been shown to provide adequate analgesia in dogs undergoing ovariohysterectomies at 10 mg/kg/hr IV (Gutierrez-Blanco et al., 2015). According to De Moura et al. (2022), fentanyl infusions at 5 mg/kg/hr IV provided adequate peri and postoperative analgesia in dogs undergoing surgical mastectomies. The quality of perioperative analgesia was considered ‘good’ in dogs receiving fentanyl at 4 mg/kg/hr IV while undergoing orthopaedic surgery, although rescue analgesia was required in 4/8 (50%) of cases (Bufalari et al., 2007).

The study by Leung et al. (2021) did not find a statistical difference between paracetamol (20 mg/kg IV) and saline. However, the study was terminated prematurely. Considering the results obtained (rescue analgesia was needed in 4/7 (57.1%) of dogs in the paracetamol group and 6/7 (85.7%) of dogs in group saline), at least 40 dogs per group would have been required to prove the superiority of paracetamol. Furthermore, paracetamol was administered postoperatively. It is unknown if its preoperative administration, at 20 mg/kg or higher doses, would have produced a better postoperative analgesic effect. However, it is probable that paracetamol alone is not a very effective analgesic in dogs undergoing ovariohysterectomy.

The pre-emptive administration of paracetamol, even at a lower dose (15 mg/kg IV), might be responsible for the better postoperative analgesia reported by Hernández-Avalos et al. (2020). This study concluded that the effect of paracetamol is equivalent to that of meloxicam and carprofen. However, plasma concentrations were not analysed. According to Leung et al. (2021), 40 minutes after the IV administration of paracetamol (20 mg/kg), its plasmatic concentration was < 10 µg/ml in all dogs. This is well below the plasma concentration that provides analgesia in humans (Gibb & Anderson, 2008; and Brett et al., 2012). Therefore, more research is warranted to better characterise the optimal dose and the analgesic activity of paracetamol in dogs. The two clinical studies used different methods to assess pain. The Hernández-Avalos et al. (2020) study used the Dynamic Interactive Visual Analogue Scale (DIVAS) and the University of Melbourne Pain Scale (UMPS), both of which are non-validated in the dog. In contrast, Leung et al. (2021) used the Short Form Glasgow Composite Pain Scale, which is a multi-item behavioural pain assessment tool developed and validated using a psychometric approach in the dog (Reid et al., 2007).

In Hernández-Avalos et al. (2020) study, one individual performed all the assessments, but it is impossible to rule out if one scale influenced the results of the second one. Therefore, both studies should be interpreted with caution.

The third study outlined in this summary (González-Blanco et al., 2020) is a well-designed study which also used a 15 mg/kg paracetamol dose similar to the Hernández-Avalos et al. (2020) study. A plasma sample was obtained 2 minutes after the administration of paracetamol and approximately 2 hours later. Similarly to Leung et al. (2021), the plasma concentrations of paracetamol falls below therapeutic levels 2 hours after its administration. The baseline minimum alveolar concentration (MAC) value of sevoflurane was determined 20 minutes post-induction of anaesthesia (MAC1), and a second MAC value (MAC2) was determined 2–2.5 hours after paracetamol or saline administration. While the MAC2 value of the paracetamol group was 15% lower than the control group, it was identical to MAC1. Instead, in the saline group, MAC2 was 15% higher than MAC1. Therefore, the difference found was not deemed clinically significant by the authors. The results of this study might be affected by the small sample size, by the use of propofol as an induction agent that could have affected MAC1 measurements, and the fact that the therapeutic plasmatic concentrations of paracetamol were potentially too low for the type of noxious stimulus applied to determine MAC2. Furthermore, this study is less relevant to the PICO question as it does not evaluate postoperative analgesia but an anti-nociceptive effect.

Despite many drugs having a MAC-sparing effect without inducing analgesia (i.e. acepromazine), analgesic drugs can have analgesic effects without producing a clinically relevant MAC-sparing effect (i.e. NSAIDs) (Reed & Doherty, 2018). According to Yamashita et al. (2008), a reduction of MAC was demonstrated by carprofen and meloxicam by 11% and 13%, respectively, similar to what was reported by González-Blanco et al. (2020).

All three reviewed studies reported no adverse side effects after a single IV paracetamol injection. In particular, Hernández-Avalos et al. (2020) did not report any change in cardiorespiratory, liver and renal parameters up to 48 hours postoperatively following 8 hourly continued IV paracetamol administered in healthy dogs. However, toxic side effects, including depression, weakness, recumbency and methaemoglobinaemia, were observed following the administration of a single dose of 150 mg/kg IV of paracetamol in dogs (St. Omer & Mohammad, 1984). While paracetamol (15–20 mg/kg IV) seems to be safe in healthy dogs, further research is required to fully evaluate its real analgesic effect, the dose at which it should be used, and the potential side effects caused by repetitive administration in both healthy and non-healthy dogs.

## Methodology

**Search strategy:** 

Databases searched and dates covered:	CAB Abstracts on OVID Platform 1975–2023 PubMed (NCBI) 1977–2024 Web of Science Core Collection 1997–2024
Search strategy:	CAB Abstracts: (dog OR dogs OR canine OR canines) AND (paracetamol OR acetaminophen) AND (intravenous OR IV) PubMed: (dog OR dogs OR canine OR canines) AND (paracetamol OR acetaminophen) AND (intravenous OR IV) Web of Science: Dog AND intravenous paracetamol
Dates searches performed:	14 Dec 2023

**Exclusion / inclusion criteria:** 

Exclusion:	Studies using oral formulations of paracetamol, pharmacokinetic studies, and narrative reviews.
Inclusion:	Systemic reviews, any comparative studies with placebo or non-steroidal anti-inflammatories drugs (NSAIDs), and any studies assessing nociception or minimum alveolar concentration (MAC) reduction.

**Search outcome:** 

Database	Number of results	Excluded – Not relevant to the PICO question	Excluded – Oral formulation research	Excluded – Narrative review	Excluded – Pharmacokinetics study	Total relevant papers
CAB Abstracts	18	11	0	0	5	2
PubMed	45	37	0	0	5	3
Web of Science	19	13	0	0	3	3
Total relevant papers when duplicates removed						3

## ORCiD

Laura Mckay: https://orcid.org/0009-0004-7079-814X

## Conflict of interest

The author declares no conflicts of interest.
